# Supplementary material for: Predicting subsurface sonar observations with satellite-derived ocean surface data in the California Current Ecosystem
Source: PLoS One. 2021 Aug 20;16(8):e0248297. doi: 10.1371/journal.pone.0248297 (PMC8378737; doi:10.1371/journal.pone.0248297)
Supplement: Attachment — Submitted filename: response_PONE-D-20-19973.pdf [file pone.0248297.s001.pdf]

Reviewer #1

This paper uses satellite and other simple environmental data to examine the statistical relationships with measures of acoustic backscatter with the goal of determining if surface satellite data can be used to forecast subsurface biological resources. In general, the statistical models with satellite data did no better (and sometimes worse) than those that did not include satellite data. I think it's worthwhile pointing out that all of the models performed quite poorly, suggesting that the right parameters were not included in the model. To that end, I have made some specific suggestions below. Developing a strong model was not the stated goal of the paper but stronger explanatory power would have provided reassurance that the general approach was valid and a stronger test of the importance of satellite observations.

I also have concerns about the writing style, particularly in the Introduction. There are no topic sentences within most of the paragraphs and transitions are weak or absent. As a result, it is really hard to identify the gaps in our knowledge and the goals of the study on the first read. I've made some specific suggestions for modifying the text to address this but these should not be considered exclusive.

> *We sincerely thank Reviewer #1 for taking their valuable time to review our manuscript and offer constructive suggestions. We have given careful consideration to each of them, as noted below.*

[1] Line 13: I think it's worth mentioning early that most ship-based echosounder systems miss the upper 5-10 m of the water column because of the depth of the hull, transducer ringdown, and other factors.

> *We now mention this additional limitation as suggested.*

[2] Line 17: The transition here is really abrupt. There is no topic sentence that connects the ideas about remote sensing to the measurements presented. What is the take away point here? It needs to be stated up front.

> *We have added a transitional sentence to provide context to the paragraph.*

[3] Line 28: Similar to above, there is not a clear connection here to previous ideas. Have remote sensing tools been used in the California Current? Why are you focusing on the CCE for this study?

> *We have added a transitional sentence to provide context, and a few example references highlighting the fact that this region has been studied extensively from the perspective of in situ measurements, satellites, and models.*

[4] Line 39: Again, there is no topic sentence. Also, I'm not sure which fish are found to 300 and which 100 m since three species are referenced in this line.

> *A topic sentence is no longer necessary as this (short) paragraph has been merged with the previous paragraph, which is a smoother transition given that the previous paragraph was to end on a reference to a previous study modeling pelagic fish in the CCE. The sentence about depth ranges has been modified to remove ambiguity about which species can be found down to 100 m vs. 300 m.*

[5] Line 48: This paragraph, with modifications, should come before the description of the CCE.

This paragraph has been moved as suggested.

[6] Line 133: I think it's a stretch from the reference to say that myctophids in the CCE reflect strongly at low frequencies. At the very least, the statement should be qualified.

The qualification "likely" has been added to the statement in question to indicate that this is our informed, professional interpretation - not necessarily a fact.

[7] 146: How much does the optical column contributing to MODIS vary?

As outlined recently by Werdell et al. (2018), this question scratches at an incredibly complex problem that we should not attempt to summarize here with a simple depth range. Except for the case of extremely clear (optically shallow) water, we can safely stand by our statement that "the shallower depths of the sonar data [are] most relevant to the satellite derived observations."

Werdel et al. (2018) An overview of approaches and challenges for retrieving marine inherent optical properties from ocean color remote sensing, Progress in Oceanography https://doi.org/10.1016/j.pocean.2018.01.001

[8] Line 136: Why was only sea surface color utilized? Why not include other satellite derived data such as sea surface height and temperature? Edited to add that I see at least SST was used as well in Table 1 but it was not included in the description of the methods.

Thank you for noticing that we used SST as well. Our choice not to use SSH was based on multiple factors such as scope, and the fact that SSH is measured by a completely different satellite platform (altimeters, which are not on board MODIS) than that/those being used in this study (MODIS). There would also be a large spatial mismatch in terms of horizontal resolution between the SSH and Chl/SST.

[9] Line 153: Instead of just including ocean depth, bottom slope or some other measurement of structure would probably be more informative.

We think this is an interesting comment. The slope, spatial variance (e.g., the Laplacian), etc. are alternative ways to characterize the seafloor, but it is doubtful that doing so would alter the conclusions of the study regarding the use of satellite data for predicting subsurface biomass. In summary, we cannot justify a redo of the whole study at this stage for one speculative (even if interesting) idea.

[10] Conclusions: I was surprised not to see any discussion of lags between chlorophyll production and fish production which could lead to the mismatches observed. It would be interesting to test this using satellite data derived from a period before the acoustic observations to see if there was a better fit. I think it also bears mentioning that previous attempts to use satellite data to predict animal distributions have largely focused on individual species (like sardine) where physiological limitations and foraging condition preferences are known.

Looking at the grab bag that NASC is mixes across those mechanisms and species, clouding the picture. In addition, Chlorophyll is a measure of standing stock, not production which further complicates the interpretation.

This is an important point. In general, the literature suggests that we might expect a lag of a few days (based on stability of oceanic fronts) for tuna-type species. As we are picking up organisms of lower trophic levels, the expected lag in our analysis might be shorter. However, as indicated in Materials and methods/Satellite data, our analysis uses 7-day averages (for a few reasons, including to account for missing pixels in the MODIS data). This averaging interval would, by coincidence, also wash out such lags and make our analysis less susceptible to "missing" the link entirely because of a lag. We have added mention of this effect to Materials and methods/Satellite data.

Reviewer #2

The manuscript Predicting subsurface sonar observations with satellite-derived ocean surface data in the California Current Ecosystem describes an attempt to predict acoustic backscatter from a diverse community of marine organisms using remotely-sensed information. From the get go, I would not expect much success on that endeavor, considering that acoustic backscatter is affected by a myriad of factors, including community structure and their sizes and anatomies, all of which are changing seasonally and annually. However, the authors' approach and analysis is sound, and one that I particularly appreciate. The cross-validation approaches utilized are adequate to test the predictive performance of the models and should be welcome in the literature. Moreover, unlike many others, the authors did not try to oversell their findings or presented conclusions not supported by the data; the speculations and suggestions into future research avenues are sensible. Overall, despite not yielding what I would consider a "positive" result, this paper stands on its good quality and would certainly serve the scientific community as a blueprint for other studies applying the same type of analyses. In conclusion, I recommend the paper to be published with minor modifications.

We sincerely thank Reviewer #2 for taking their valuable time to review our manuscript and offer constructive suggestions. We have given careful consideration to each of them, as noted below.

Minor comments:

[1] Line 31-33: "Aggregations of euphausiid species in the CCE, namely North Pacific krill (Euphausia pacifica), are typically found less than 30 m depth during the night and between 50-100 m depth…" Thysanoessa spinifera is very abundant too in the CCE, and there are many other minor species that could be important at a local level.

We now mention the abundance of Thysanoessa spinifera and other species that may be important at a local level.

[2] Line 82: "On-axis [7] beam-pattern measurements were also taken." Please remove or rephrase. Beam pattern refers to the sensitivity of transmission and reception as a function of angle beyond the axis.

The sentence has been removed as suggested.
