# Supplementary material for: Predicting subsurface sonar observations with satellite-derived ocean surface data in the California Current Ecosystem
Source: PLoS One. 2021 Aug 20;16(8):e0248297. doi: 10.1371/journal.pone.0248297 (PMC8378737; doi:10.1371/journal.pone.0248297)
Supplement: Attachment — Submitted filename: 2020-02-18-plos-one-sonar-response.pdf [file pone.0248297.s002.pdf]

Dear Editor,

Thank you for taking the time to review our manuscript and suggest changes to improve the impact of the paper. Below, we address each point in turn with our responses in italics.

1. You state: 'Additionally, there was potential for geographic inaccuracies in the aggregation of the sonar data to the spatial resolution of the MODIS product. The sonar data are recorded and stored as irregular point data, and when aggregated to 4 km to match the MODIS products there was no guarantee that the aggregation window matched exactly that of a single MODIS grid cell.'

Can you please elaborate?
I believe the georeferencing of MODIS is quite good as well as those from your GPS.
Do you imply a problem with the fact that the time measurements were taken at is not the same as the satellite pass which may result in advection of features? If yes, please say so clearly.
The way your explanation is written suggests that maybe it is an issue of subgrid variability (satellite product you use average a 4x4km piece of ocean). Do you have evidence of such patchiness?
BTW, you can obtain chlorophyll at Level 2 at 1x1km grid. No reason to limit yourself to Level 3.
You can also use inline sensors, if available, to assess sub-grid variability in some properties.

Thanks for raising this issue. We are referring to the potential for spatial misalignment between the MODIS grid and the 4km track aggregation that was performed on the sonar data. Both data products are at 4km, but there are no guarantees that the boundaries align, e.g., a 4km stretch of the vessel's path need not necessarily begin and end at the boundary of a 4km MODIS pixel. We have clarified in text as follows:

"Additionally, there was potential for geographic misalignment in the spatial aggregation of the sonar data to the spatial resolution of the MODIS product. The sonar data are recorded and stored as irregular point data, and when aggregated to 4 km horizontal bins along the vessel's path, there is no guarantee that this aggregation along the path matches the MODIS grid. In other words, while both data products are aggregated to 4m, the spatial boundaries between 4km sonar bins and 4km MODIS grid cells were not perfectly aligned."

2. You do not mention ship noise as a possibility for surface acoustics not matching. Did you use a 'quiet' vessel? Is there reason to believe that there may be a response to the vessel's noise? I am not an acoustician and since the reviewers have not raised this issue, it is only a comment.

The NOAA FSV Bell M. Shimada is a quiet vessel. The ship's design allows for quieter operation and movement of the vessel through the water, allowing scientists to study fish and marine mammals without disturbing them. The quietness is focused towards reducing low frequency sounds that would be noticeable in passive acoustic research. There is perhaps also

a reduction in bubbles produced during transit that could be picked up in the near field of the sonar. However, the first 10 m of the acoustic data are removed from analysis to reduce the influence of any bubbles and transducer ring down so the quiet rating of the vessel wouldn't impact the matching or lack thereof surface acoustics. Our paper states: " To minimize the effect of surface bubbles and transducer ringdown, acoustic data were collected from 10 m below the surface, roughly 5 m below the centreboard-mounted transducer face." There is no need to add additional text.

3. Please do not call the z1% product 'euphotic depth'. It is an anachronism (I know NASA still uses that, but not for much longer).
Primary productivity is limited by the absolute intensity of light, not relative to a surface value that changes throughout the year. Keep it as the 1% light level. People will know exactly what you mean.

Thanks for the suggestion - we have changed “euphotic depth” to “1% light level zone depth”.
